# Automated Segmentation of Nuclei in Breast Cancer Histopathology Images

**DOI:** 10.1371/journal.pone.0162053

**Published:** 2016-09-20

**Authors:** Maqlin Paramanandam, Michael O’Byrne, Bidisha Ghosh, Joy John Mammen, Marie Therese Manipadam, Robinson Thamburaj, Vikram Pakrashi

**Affiliations:** 1 Department of Mathematics, Madras Christian College, Chennai, India; 2 School of Mechanical and Materials Engineering, University College Dublin, Ireland; 3 Department of Civil, Structural and Environmental Engineering, Trinity College Dublin, Ireland; 4 Department of Transfusion Medicine & Immunohematology, Christian Medical College, Vellore, India; 5 Department of Pathology, Christian Medical College, Vellore, India; Fu Jen Catholic University, TAIWAN

## Abstract

The process of Nuclei detection in high-grade breast cancer images is quite challenging in the case of image processing techniques due to certain heterogeneous characteristics of cancer nuclei such as enlarged and irregularly shaped nuclei, highly coarse chromatin marginalized to the nuclei periphery and visible nucleoli. Recent reviews state that existing techniques show appreciable segmentation accuracy on breast histopathology images whose nuclei are dispersed and regular in texture and shape; however, typical cancer nuclei are often clustered and have irregular texture and shape properties. This paper proposes a novel segmentation algorithm for detecting individual nuclei from Hematoxylin and Eosin (H&E) stained breast histopathology images. This detection framework estimates a nuclei saliency map using tensor voting followed by boundary extraction of the nuclei on the saliency map using a Loopy Back Propagation (LBP) algorithm on a Markov Random Field (MRF). The method was tested on both whole-slide images and frames of breast cancer histopathology images. Experimental results demonstrate high segmentation performance with efficient precision, recall and dice-coefficient rates, upon testing high-grade breast cancer images containing several thousand nuclei. In addition to the optimal performance on the highly complex images presented in this paper, this method also gave appreciable results in comparison with two recently published methods—Wienert et al. (2012) and Veta et al. (2013), which were tested using their own datasets.

## 1. Introduction

Breast Cancer is the most prevalent type of cancer in women worldwide [1]. Current breast cancer clinical practice and treatment mainly relies on the evaluation of the disease’s prognosis. A semi-quantitative assessment of the breast cancer prognosis is well established by the Bloom-Richardson grading system [2] which defines the scoring of three morphological features of the suspicious tissue: 1) percentage of tubule formation, 2) degree of nuclear pleomorphism, and 3) mitotic cell count. The scoring is done based on a pathologist's visual examination of the biopsy specimen of the tissue under microscope which has a substandard reproducibility [3]. In order to mitigate this issue and provide quantitative reproducible parameters researchers have suggested the use of Image Analysis methods [4]. For instance, [2] had suggested the use of image analysis of the breast histology tissue for accurate estimation of nuclei size and shape differences. With relevance to these propositions, the advancements in digital pathology [5] and the advent of fast digital slide scanners [6] had simplified the digitization of histopathology slides and opened the possibility to apply image analysis techniques. Histopathology image datasets are available online from various open sources, such as the UCSB dataset from Center for Bio-Image Informatics, University of California, Santa Barbara [7], MITOS-ATYPIA grand challenge dataset [8], and the Assessment of Mitosis Detection Algorithms (AMIDA13) dataset [9].

**Fig 1:**
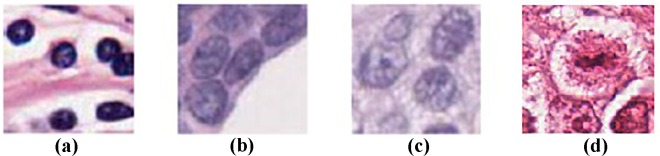
a) Lymphocyte (LN), b) Normal Epithelial nuclei (EN), c) Cancerous Epithelial Nuclei (CN) and d) Mitotic nuclei (MN)

Automated segmentation of nuclei is the most crucial step in quantitative image based analysis of breast histopathology and has remained challenging due to the complex appearance of the tissue. Reviews state that the proposed segmentation frameworks in literature have poor segmentation accuracy for images containing epithelial cancerous nuclei (CN) especially when CN are clustered and overlapping. In addition, the traditional techniques are intolerant to other forms of CN which range from round-like shaped normal nuclei to large irregularly shaped nuclei with highly coarse chromatin marginalized to the nuclei periphery and occasionally marked by the presence of a prominent nucleoli. Breast histopathology images may also contain other objects like lymphocyte nuclei (LN) and occasional stain-artifacts which may affect the specificity of the algorithms which aim at detecting just CN alone. Fig 1 shows the different nuclei types of nuclei which are of interest in breast histopathology images. The Hematoxylin and Eosin (H&E) staining of the slides, which is the standard staining protocol, is used in breast histopathology tissue preparation and hence the nuclei are blue colored and stromal tissue are pink colored.

Given the importance and challenges of segmenting cancerous nuclei in breast histopathology images, this paper proposes a novel segmentation framework that implements tensor voting followed by Loopy Belief Propagation (LBP) on a Markov Random Field (MRF) for nuclei delineation in breast cancer histopathology images. Tensor voting is more efficient than traditional clustering techniques in that it is a powerful salient-feature estimator as it comes with the ability to encode magnitude and orientation simultaneously. Herein the tensor voting is done in the direction of image gradient to detect nuclei seed points and then an MRF driven Loopy back propagation algorithm is used to derive nuclei boundaries. The target of the proposed methodology is to provide a better detection rate for cell-images with normal or low grade cancer and to create a benchmark of detection for the more difficult images of high grade cancer cells for which little detection-related information is available at present in published literature. The paper is organized as follows: Section 2 gives a short review of the related works in literature. Section 3 presents the dataset and ground truth followed by the methodology and the results are explained in Section 4 and Section 5 respectively. At the end, the concluding remarks are discussed in Section 6.

## 2. Related Works

Numerous authors have proposed different methods for breast histopathology nuclei segmentation with each of them using certain segmentation technique such as thresholding, morphological operations, watershed, active contour models, and G-cuts either separately or in combination [10]. The methodologies vary not only in their segmentation techniques but also in the approach towards nuclei detection steps.

One approach is finding a seed point within each nuclei region and then deriving the boundary of the nuclei initializing at the seed point. [11] had proposed the use of Hough transform technique for detecting nuclei seed points which were used in initializing a shape- and texture-based active contour model. In [12] nuclei seed points are found by the peaks of Euclidean distance map and then the image is transformed to gradients in polar space (GiPS) by converting nuclei pixels co-ordinates to polar co-ordinates having the seed points as the origin. A bi-quadratic filtering is then applied to the resultant gradient image to obtain the nuclei boundaries. A few authors have presented different voting algorithms which cast votes along gradient directions amplifying votes inside the centre of nuclei thereby locating the seed points as ones having maximum votes [13], [14] and [15]. Subsequently the detected nuclei seed points were either used to initialize active contours [13] or an edge grouping algorithm [15]. [16] applied the marker-controlled watershed approach at multiple scales, the segmentation looks into nuclei of all sizes. Two types of markers were proposed, one using radial symmetry transform (RST) and the other, the regional minima of the pre-processed image. Size, shape, boundary, chromatin distribution features and solidity of the object are all used in determining if an object is a valid nuclei or not.

Another approach is to segment the nuclei regions and then resolve the overlapping or clump nuclei separation through heuristic approaches like the Concave Point Detection [17]; [18]. In [17] a magneto-static active contour model initialized by Expectation maximization (EM) based binarization, provides a force which guides the contours to object boundaries. [18] has contributed a novel contour-based “minimum-model” segmentation approach that uses minimal a priori information and detects contours independent of their shape. [19] presented an integrated region, boundary and shape based active contour to handle nuclei, lymphocytes and gland segmentation in H&E stained prostate and breast histopathology images.

This section consists of a short list of methodologies proposed for segmentation of cancerous epithelial nuclei which is the scope of this paper. However methods have been proposed for detecting other objects like lymphocytes [20], and mitotic cells ([8]; [21]), which are considered as special cases, in breast histopathology images. A consolidated review of the several issues on breast cancer histopathology image analysis can be found [22]. A detailed review of the histopathology nuclei detection, segmentation and classification methods can be found in [10].

## 3. Dataset and Ground Truth Data

The proposed methodology was tested and evaluated on de-identified and de-linked images of histopathology specimens from the Department of Pathology, Christian Medical College Hospital (CMC),The proposed method was validated on eight representative images of H&E stained breast cancer histopathology sections. These were images of regions afflicted by tumor growth captured from biopsy slides of different patients through a digital camera, Leica DFC280, attached to a compound microscope setup at x40 magnification. All the images were annotated by the pathologist who provided a nuclear pleomorphism score based on the Bloom Richardson protocol. The images had dimensions of 1024 × 1280 pixels. The pathologist set a score of 3 for images showing marked variation in nuclei-size and appearance upon comparison with normal cells, reflecting the presence of high grade cancer cells, a score of 2 for moderate variations and a score of 1 for mild variations. In addition to these images, two Whole Slide Images (WSI) of H&E stained breast biopsy slides diagnosed for invasive ductal carcinoma were used in the study. The dimensions of the imaged were 80784 x 148672 pixels captured at x40 magnification by a Ventana slide scanner and stored in the BIF format. The de-identified and de-linked images were collected with the informed consent by review and approval of the Institutional Review Board (Silver, Research and Ethics committee) of the Christian Medical College, Vellore. Moreover the proposed method was evaluated and compared on three different datasets from recent research articles: [18] and [16]. [18] dataset consists of Hematoxylin-Eosin stained histopathology images from breast, liver and bone marrow and other tissues each of which was 600×600 pixels in size. The [16] training dataset consisted of 21 images from different breast histopathology slides and the testing dataset consisted of 18 images from different breast histopathology slides. The authors developed the segmentation procedure on training dataset and validated the procedure on testing dataset. The results of the proposed method for these datasets have been discussed and compared with the work of their corresponding authors in Section 5.

## 4. Methodology

**Fig 2:**
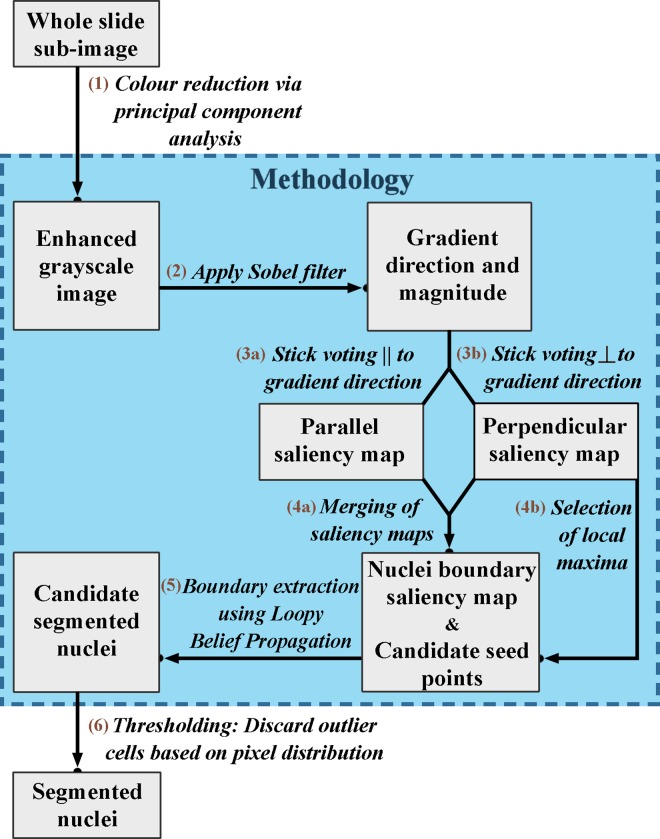
Flowchart for the proposed nuclei segmentation technique.

An overview of the technique is illustrated in the flowchart in Fig 2.

The segmentation framework can be divided into four main steps: 1. Pre-processing, 2. Nuclei saliency map construction, 3. Nuclei boundary extraction and 4. Post-processing.

### 4.1 Pre-processing and gradient computation

**Fig 3:**
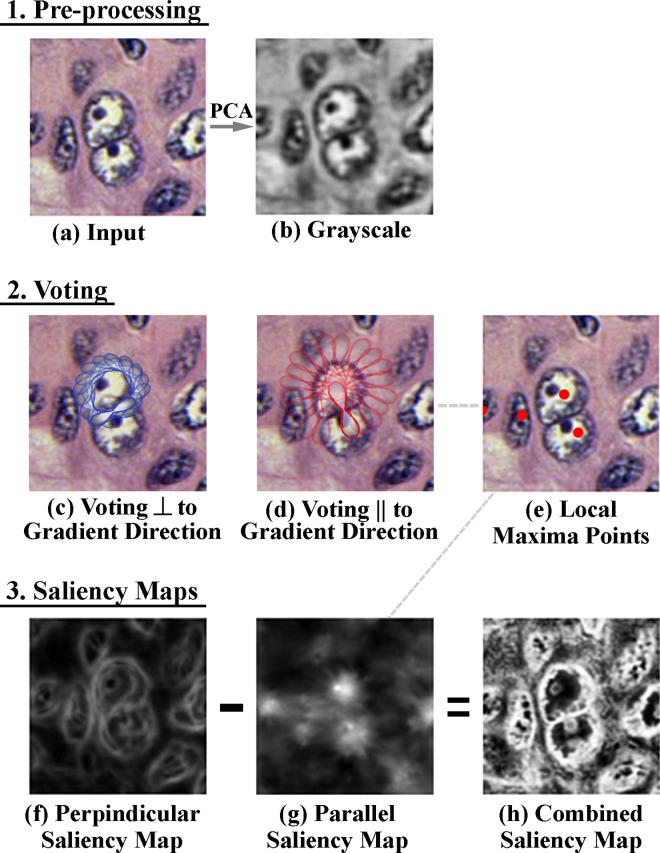
a) A Sub Image showing an overlapping nuclei cluster, b) pre-processing: enhanced grayscale image which is obtained by applying principal component analysis to image (a), c) Graphical illustration of stick tokens oriented perpendicular to gradient direction. d) Graphical illustration of stick tokens oriented parallel to gradient directions. e) Nuclei seed points plotted in red–found using non regional maximal suppression of the parallel saliency map f) result of parallel voting g) result of perpendicular voting, h) combined nuclei saliency map obtained by subtracting (g) from (f).

The input RGB image is reduced to a 2D intensity image using principle component analysis (PCA). PCA was employed as it was found to be an effective way of concisely representing color information, and it worked well irrespective of variations in the level of H&E staining in the input images. A sub-image of the original input RGB image is shown in Fig 3A, which depicts an overlapping nuclei cluster, and the corresponding PCA-reduced grayscale image is shown in in Fig 3B. The gradient magnitude, *G*, and direction, *α*, are computed for this grayscale image, denoted by A, by convolving it with the Sobel operator, as per:

$$\begin{array}{ccc} G_{H}=\left[\begin{array}{ccc} -1 & 0 & +1\\ -2 & 0 & +2\\ -1 & 0 & +1 \end{array}\right]*A & & G_{V}={\left[\begin{array}{ccc} -1 & 0 & +1\\ -2 & 0 & +2\\ -1 & 0 & +1 \end{array}\right]}^{T}*A \end{array}$$ (1)

$$G=\sqrt{G_{H}{}^{2}+G_{V}{}^{2}}$$ (2)

$$\alpha =\tan^{-1}\left(\frac{V}{H}\right)$$ (3)

where *G*_*H*_ is the horizontal gradient image and *G*_*V*_ is the vertical gradient image. The gradient information is used to orientate and weight the votes in the next phase of this algorithm.

### 4.2 Nuclei saliency map construction using tensor voting

Given a description of the complex appearance of cancerous nuclei as having an irregular shape, coarse chromatin and visible nucleoli, at this point in the algorithm, we incorporate certain known cues that the nuclei are rounded structures with sharp intensity gradient at their boundaries. Hence a tensor voting framework [23] is used to perform voting along the image gradient directions. The voting process is useful for highlighting salient features, such as the nuclei boundaries and centers, while suppressing background pixels.

A standard stick voting approach is employed which relies on a set of short voting sticks to propagate information to nearby pixels. The voting field, *DF*, for a stick centered at a point (*x*,*y*) in the image is defined using a decay function. This function which is weighted by the gradient magnitude, *G*_*xy*_ and the image intensity, *A*_*xy*_, (or more correctly, 1 –*A*_*xy*_) at that point, This is to capitalize on the fact that nuclei boundaries tend to be darker than their surroundings and they correspond to points in the image where there is a sharp intensity gradient is present. Weighting in such a manner gives greater prominence to votes that coalesce at the nuclei boundaries. This serves to amplify the edge boundaries when the votes are orientated perpendicular to the gradient, and it serves to highlight the center of nuclei when the votes are orientated parallel to the image gradient. For voting parallel to the image gradient, the decay function is given by:

$$\text{DF}{\left(\text{s},\text{k},\sigma \right)}_{xy}={\text{e}}^{-\left(\frac{{\text{s}}^{2}+{\text{ck}}^{2}}{\sigma^{2}}\right)}G_{xy}(1-A_{xy})$$ (4)

where, $s=\frac{\alpha_{xy}l}{\sin(\alpha_{xy})}$ is the arc length from the voting stick (voter) to a target location in its voting field (receiver), *l* is the length between the voter and the receiver, $k=\frac{2\;\sin(\alpha_{xy})}{l}$ is the curvature, $c=\frac{-16log(0.1)(\sigma -1)}{\pi^{2}}$ controls the degree of decay with curvature and σ is the scale of the voting stick, which determines the neighbourhood size. Voting perpendicular to the image gradient direction is carried out in the same fashion, with the only changebeing that $s=\frac{-\alpha_{xy}{}^{-1}l}{\sin(-\alpha_{xy}{}^{-1})}$ and $k=\frac{2\;\sin(-\alpha_{xy}{}^{-1})}{l}$ to reflect the change in angle. The shape of a typical voting field can be observed in Fig 3C and 3D, which show the voting sticks and their associated fields applied perpendicular and parallel to the image gradient, respectively. At this point, candidate nuclei seed points are found by applying a non-regional maxima suppression algorithm to the parallel saliency map as described by [24]. The detected candidate seed points for this illustrated sub-image are shown in (Fig 3E).

**Fig 4:**
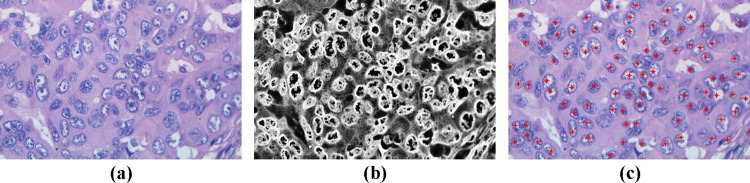
a) Sub Image of a breast histopathology section b) Nuclei saliency map and c) Nuclei seed points

At each target location (point in the image) the voting fields from all contributing voting sticks are added up to arrive at a saliency map. A sample saliency map corresponding to the perpendicular-applied votes is illustrated in Fig 3F, while a saliency map for the parallel-applied votes is shown in Fig 3G. A combined saliency map, which is used in the subsequent boundary extraction phase, is formed by subtracting the parallel saliency map from the perpendicular saliency map, as shown in Fig 3H. It may be noted that the nuclei boundaries in the combined saliency map appear particularly accentuated, even for vague cells, which underlines the efficiency of this voting framework. Fig 4 shows the outcome of this procedure on a sub-image of a breast histopathology section.

### 4.3 Nuclei boundary extraction by loopy belief propagation on a Markov random field

**Fig 5:**
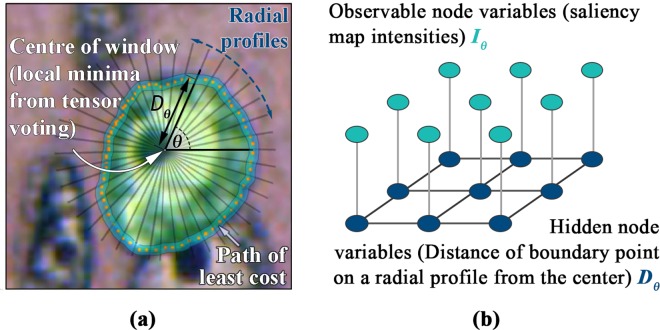
a) Graphical illustration of boundary search paths. b) MRF formulation of the boundary delineation problem.

This stage operates on the combined saliency map obtained in the previous stage of the algorithm. A window is centered at each seed point location in the combined saliency map. This window confines the search space for the nuclei boundary. The window has a predefined size; it should be chosen such that it encompasses the whole cell nuclei. It was conservatively taken to be 30×30 pixels for the images illustrated in this paper. Within each window, the most likely nuclei boundary is determined using a set of radial profiles of equal arc length intervals radiating from the center towards the edge of the window, as shown Fig 5A.

The problem is formulated based on the assumption that the boundary is a smoothly evolving and closed path, and as such, adjacent radial profiles would be expected to intersect the nuclei boundary at similar offsets from the seed point. In order to enforce boundary smoothness, the maximum allowable deviation between two radial profiles is set to be less than 5 pixels. The boundary search process is performed once for every candidate nucleus. The outcome of the procedure for one candidate nuclei is graphically depicted in Fig 5A.

**Fig 6:**
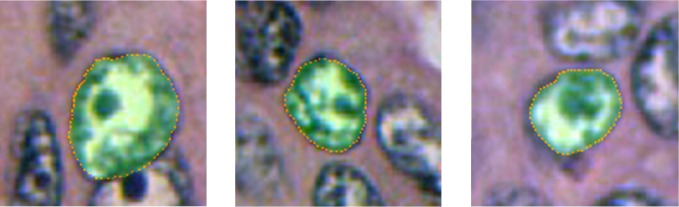
Results of boundary detection.

The neighboring/ spatial and contextual dependencies between the adjacent radial profiles make this boundary extraction problem suitable for MRF assumption. The MRF formulation is as follows: First the pixels in the search path are represented by polar co-ordinates *δ*(*θ*,*ϕ*) where *θ* is the angle representing the direction of a radial profile emanating from centre towards the window and *ϕ* is the distance of the pixels on the radial profile. The MRF observable node variables are the intensity values from the nuclei saliency map (Fig 4B) in polar co-ordinate form, and the hidden node variables are nuclei boundary points on the radial profiles that we are trying to find (shown in Fig 5B). Fig 6 shows results of boundary extraction step on sub-images containing nuclei.

The message passing between the nodes is implemented by a min-sum implementation of a LBP algorithm as follows.

Message passing is carried out across the hidden node network, whereby neighboring nodes share information with each other concerning the likelihood of a cell boundary existing at a distance, *ϕ*, along radial profile from the nuclei seed center. The MRF energy function to find the optimal boundary point *D* on a radial profile *I* is given by:

$$Ε\left(I,L\right)=\underset{i}{\sum }{Ε}_{data}\left(I_{i},l_{i}\right)+\underset{j\epsilon N_{i}}{\sum }{Ε}_{smoothness}\left(l_{i},l_{j}\right)$$ (5)

Where E(*I*,*L*) is minimum cost for finding the nuclei boundary pixel along the set of radial profiles. Here *I*_*i*_ is the intensity of pixel *i* on radial profile *I* and *j* are the pixels in the adjacent radial profiles. The first term is the data cost, that is the cost associated with keeping low the intensity discrepancies inside the nucleus and the second term is the smoothness cost ensuring no adjacent radial profile *I* should have boundary point differing by a distance of more than 5 pixels from the center.

- LBP Message update: The message from node i to j is defined by

$$msg_{i\to j}\left(l\right)=\begin{array}{c} \min\\ l^{\prime }\in X \end{array}\left[{Ε}_{data}\left(I_{i},l^{\prime }\right)+{Ε}_{smoothness}(l,\overset{´}{l^{'}}\right]+\underset{k\in N_{i}\;and\;k\neq j}{\sum }msg_{i\to j}\left(l^{\prime }\right)$$ (6)

*X* is the set of values giving the distance of pixels in adjacent radial profile from the centre of the search window.

- LBP Message initialization: All the messages (possible radii of spokes) are initialized to 0.
- LBP Belief: $Belief\;(D_{i}=l)={Ε}_{data}(I_{i},\overset{´}{l})+\sum_{k\in N_{i}}{msg}_{k\to i}(\overset{´}{l}).$ The belief (lowest cost) will yield the best possible boundary point for each radial profile emanating from the center. The resulting edge boundary of few scenes in a high grade cancer breast histopathology is shown in Fig 6.

### 4.4 Post-processing: removing spurious nuclei

While most of the segmented regions obtained from the LBP phase will likely correctly correspond to nuclei, there may also be some erroneous regions which must be filtered out. Due to the noisy and heterogeneous nature of high grade cancer images, the uncertainties of measurement reach a high level. It is thus necessary to resort to statistical based estimates as a final classification step so that valid segmented nuclei can be retained while innocuous spurious regions, such as stains can be discarded. This is achieved by computing the mean of the pixel intensity distribution within each segmented region. As the overall intensity of nuclei tends to be darker then the background pixels, regions with a mean value above some predefined threshold are assumed to be false alarms and are rejected from the final segmentation as a result. The mean of pixel values inside a detected boundary are now computed. The spurious objects are discarded by varying the mean intensity threshold because ink stains and other artifacts have a very high mean intensity values.

## 5. Experimental Results

The proposed segmentation framework was implemented using MATLAB 2013a and evaluated for images from our dataset and for images from datasets published in Wienert et al. 2012 and Veta. et al. 2013. Three important measures namely: Precision, Recall and Dice Coefficient were used to evaluate the performance of the segmentation algorithm. Here the precision measure (Positive predictive value) refers to the ratio of the number of true nuclei Table 1. Performance Analysis on Breast cancer histopathology images of various grades (manually labeled) detected among the total number of automatically detected objects. Recall (True positive rate) refers to the ratio of manually labeled nuclei that are picked by the algorithm. Both the precision and recall measures help in measuring the effectiveness of the algorithm in detecting relevant nuclei regions. The Dice coefficient gives the segmentation accuracy by measuring the dice similarity between the automatically segmented nuclei and manual segmentation. Dice coefficient of two images regions *M* and *N* is given by

$$\text{Dice Coefficient}=\frac{2\times (M\cap N)}{\left(M+N\right)}$$ (7)

**Table 1:** Performance Analysis of Proposed Method.

Image Id	Nuclear Pleomorphism Score	Number of manually segmented nuclei	Precision	Recall	Dice Coefficient
1	3	461	0.8894	0.7884	0.8180
2	3	530	0.8787	0.7360	0.8582
3	3	512	0.9657	0.7480	0.8830
4	3	610	0.9562	0.7103	0.7890
5	3	570	0.9231	0.7691	0.8400
6	1	396	0.9477	0.8335	0.8712
7	1	412	0.9192	0.8287	0.8702
8	1–2	436	0.9422	0.7236	0.8910

**Fig 7:**
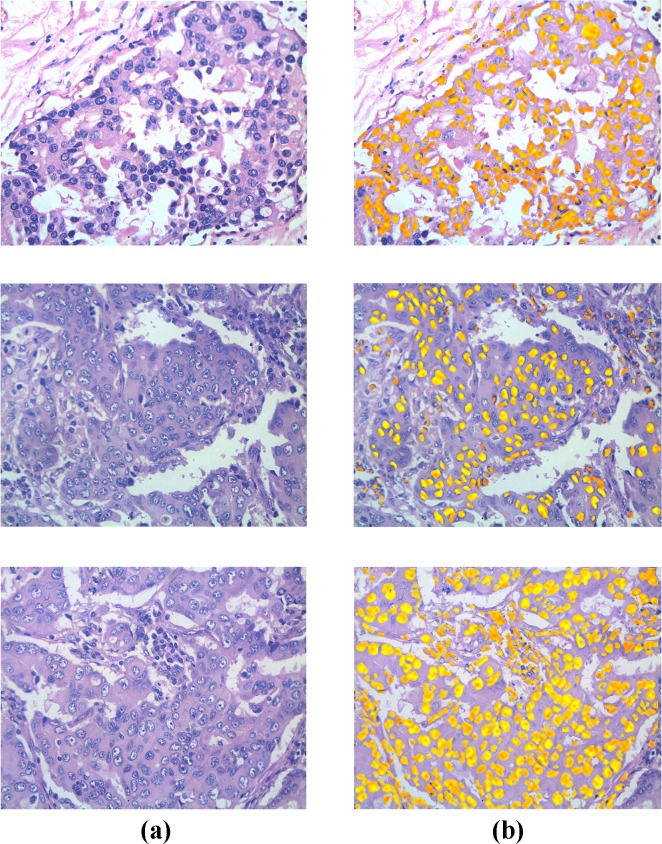
a) Original Image of grade 3 breast cancer histopathology sections, b) Corresponding segmentation results.

**Fig 8:**
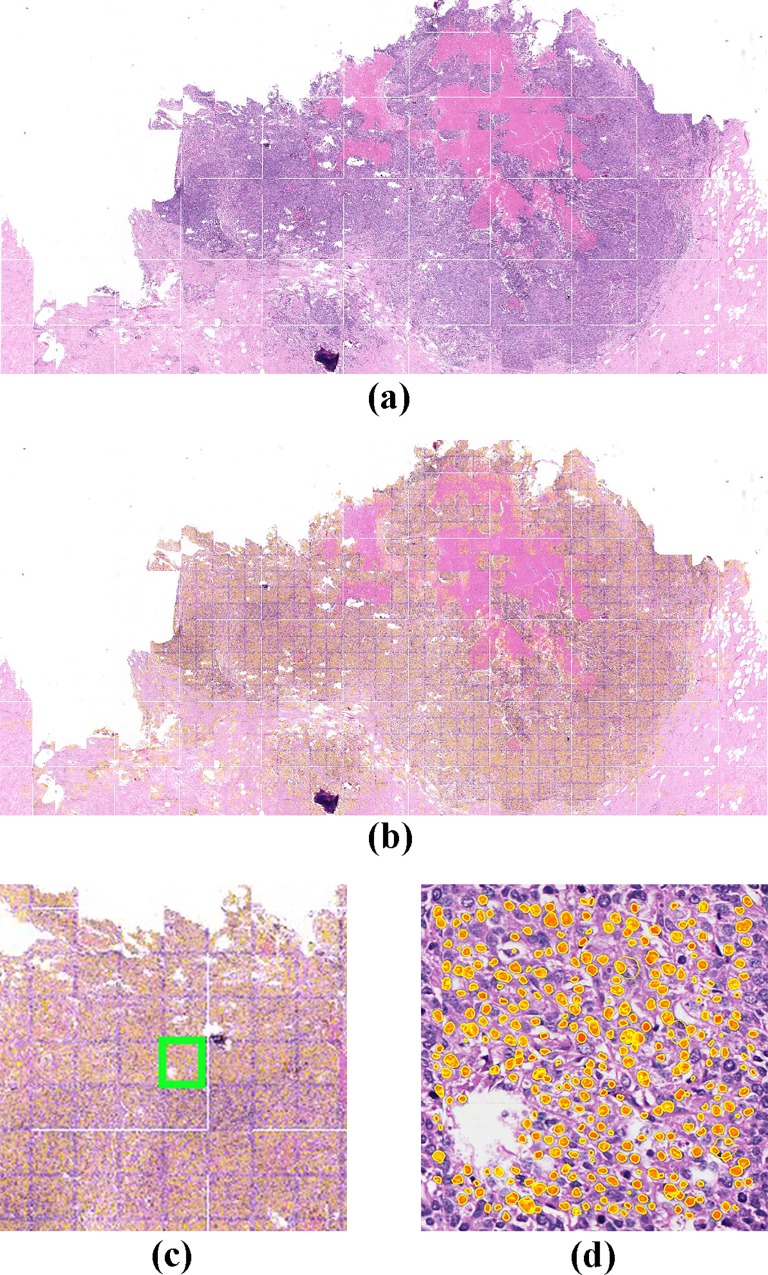
a) WSI Image of a breast cancer histopathology slide, b) Segmentation result shown in yellow, c) Shown in green box is a 1000 x 1000 pixel patch selected starting at pixel position (12000, 15000)) and d) Closer view of the segmentation result on the region selected.

The performance measures for 8 breast histopathology images in our dataset are given in Table 1. These images were selected as candidates to represent difficult-to-detect images due to their relatively huge number of cancer cells. Fig 7 illustrates the results of our algorithm when applied to a selection of these grade 3 breast cancer histopathology images, while Fig 8 showcases the results when applied to a Whole Slide Image (WSI).

**Fig 9:**
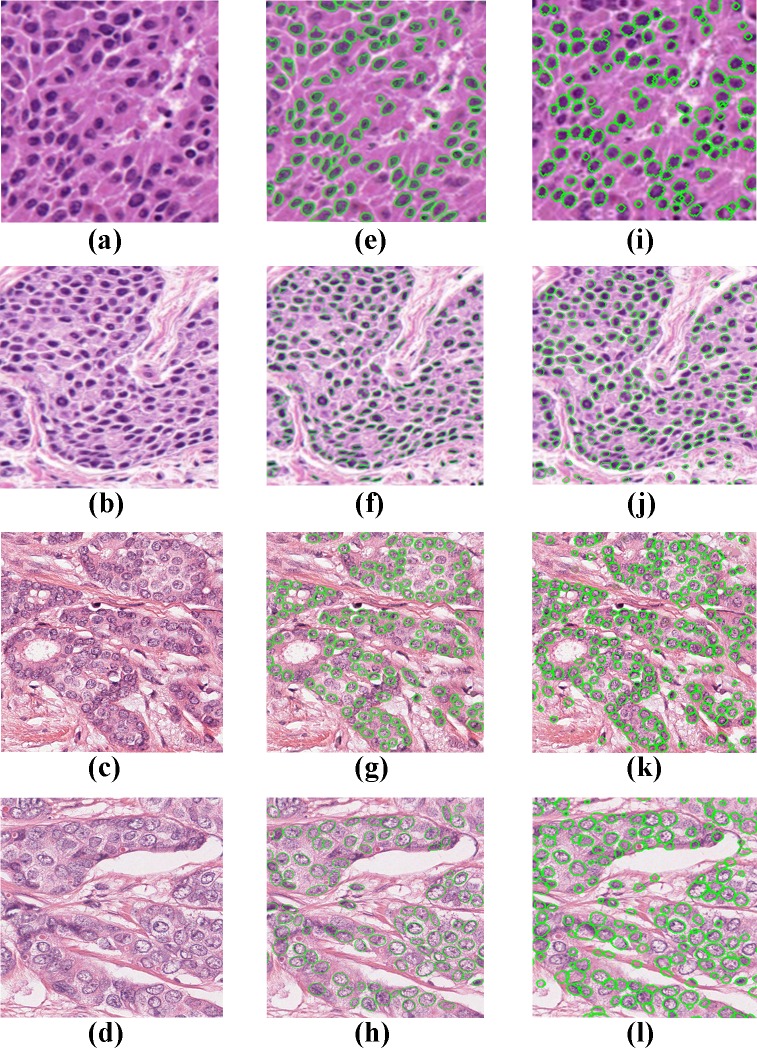
Qualitative results (A)-(D) Original Images of Breast cancer Histopathology Images, (E)&(F) Segmentation result of Wienert et al. (2012), (G)&(H) Segmentation result of method Veta. et al. (2013), (I)-(L) Segmentation results of proposed method.

The performance results of our algorithm for images from the other datasets are shown in Table 2. Herein, the ground truth information and evaluation methods were followed as given in these published articles. In addition to giving good detection and segmentation accuracy rates for the difficult-to-detect images in our dataset, the proposed method gave better results on datasets used in other studies. Fig 9 depicts segmentation results of our proposed algorithm in comparison to methods of Wienert et al. (2012) and Veta et al. (2013).

**Table 2:** Performance evaluation and comparison of the proposed method on datasets from Wienert et al. (2012) and Veta. et al. (2013)

Results	Wienert, et al. 2012	TV-MRF-BP on Wienert et al. 2012 dataset	Veta, et al. 2013 Training set	TV-MRF-BP on Veta, et al. 2013 Training set	Veta, et al. 2013 Testing set	TV-MRF-BP on Veta, et al. 2013 Testing set
No of manually counted/segmented nuclei	7831	7831	2093	2093	2191	2191
Precision	0.908	0.921	0.853	0.801	0.875	0.930
Recall	0.859	0.901	0.886	0.823	0.904	0.966
Dice Coeff			0.9	0.84	0.9	0.9

## 6. Conclusion

The proposed segmentation framework has integrated a gradient driven voting mechanism using 2D tensor voting and an MRF loopy back propagation technique to segment the individual nuclei from breast histopathology images. Test results show that the proposed method is suitable for nuclei segmentation in high-grade breast cancer histopathology images containing scenes depicting grade 3 nuclear pleomorphism (cancerous nuclei with marked variations from normal nuclei) even though these are quite challenging for traditional segmentation methods to detect. In addition, the method was tested on images from published datasets from [18] and [16] and provided better segmentation performance.

## Supporting Information

S1 File IRB.pdf (PDF)
