# Supplementary material for: Automated Segmentation of Nuclei in Breast Cancer Histopathology Images
Source: PLoS One. 2016 Sep 20;11(9):e0162053. doi: 10.1371/journal.pone.0162053 (PMC5029866; doi:10.1371/journal.pone.0162053)
Supplement: S1 File — (PDF) [file pone.0162053.s001.pdf]

OFFICE OF RESEARCH
INSTITUTIONAL REVIEW BOARD (IRB)
CHRISTIAN MEDICAL COLLEGE, VELLORE, INDIA

Ethics Committee Registration No: ECR/326/INST/TN/2013 issued under Rule 122D of the Drugs & Cosmetics Rules 1945, Govt. of India

Dr. George Thomas, D Ortho Ph.D.
Chairperson, Ethics Committee

Dr. B. Antonisamy, M.Sc., Ph.D., FSMS, FRSS.
Secretary, Research Committee

Prof. Keith Gomez, B.Sc., MA (S.W), M.Phil.
Deputy Chairperson, Ethics Committee

Dr. Alfred Job Daniel, D Ortho MS Ortho DNB Ortho.
Chairperson, Research Committee & Principal

Dr. Biju George, MBBS., MD., DM
Deputy Chairperson,
Secretary, Ethics Committee, IRB
Additional Vice-Principal (Research)

May 25, 2016

To Whom It May Concern

The Institutional Review Board (Silver, Research and Ethics Committee) of the Christian Medical College, Vellore, reviewed and discussed your software development project with de-identified and de-linked images and there are no ethical issues involved. The IRB has not objection to scientific publication of the specified work.

Dr. Biju George
Secretary (Ethics Committee)
Institutional Review Board

Dr. BIJU GEORGE
MBBS., MD., DM.
SECRETARY - (ETHICS COMMITTEE)
Institutional Review Board,
Christian Medical College, Vellore - 632 002.

Ethics Committee Silver, Office of Research, I Floor, Carman Block, Christian Medical College, Vellore, Tamil Nadu 632 002
Tel: 0416 – 2284294, 2284202 Fax: 0416 – 2262788 E-mail: research@cmcvellore.ac.in
